# Local Recurrence and Metastasis Define Distinct Recurrence Phenotypes in Soft Tissue Sarcoma

**DOI:** 10.3390/cancers18132100

**Published:** 2026-06-28

**Authors:** Markus Schärer, Philip Heesen, Gabriela Studer, Bettina Vogel, Georg Schelling, Bruno Fuchs

**Affiliations:** 1 Sarcoma Service, Department of Orthopedics and Trauma, Kantonsspital Winterthur, 8400 Winterthur, Switzerland; 2 Department of Shoulder and Elbow Surgery, Schulthess Clinic Zurich, 8008 Zurich, Switzerland; 3 Medical Faculty, University of Zurich, 8032 Zurich, Switzerland; 4 Faculty of Health Sciences & Medicine, University of Lucerne, Frohburgstrasse 3, 6002 Luzern, Switzerland; 5 Sarcoma Service, Department of Radiation Oncology, Sarcoma Center, LUKS University Hospital, 6000 Lucerne, Switzerland; 6 Sarcoma Service, Department of Orthopedics and Trauma, Sarcoma Center, LUKS University Hospital, 6000 Lucerne, Switzerland

**Keywords:** soft tissue sarcoma, local recurrence, distant metastasis, recurrence patterns, disease progression, prognostic factors, tumor grade, real-world data, sarcoma registry

## Abstract

**Background**: Soft tissue sarcomas (STS) are clinically heterogeneous, yet local recurrence (LR) and metastasis are typically analyzed as separate endpoints. We aimed to determine whether recurrence in STS is better understood as distinct phenotypes defined by the interaction between local and metastatic disease. **Methods**: A total of 668 patients with histologically confirmed STS from two tertiary sarcoma centers were analyzed using prospective registry data (2018–2025). Patients were stratified into four predefined recurrence phenotypes: no event, metastasis only, LR only, and combined LR and metastasis. Tumor characteristics, treatment variables, surgical factors, recurrence timing, and event sequence were analyzed across groups. **Results**: Phenotypes were distributed as follows: no event (52.2%), metastasis only (23.8%), LR only (13.5%), and combined recurrence (10.5%). High-grade tumors (G3) were more frequent in metastasis-only (73.0%) and combined phenotypes (65.7%) than in LR only (38.9%) and no event (28.9%) (*p* < 0.001). Early LR (≤365 days) occurred substantially more often in the combined phenotype (51.4%) than in isolated LR (17.8%), whereas late LR predominated in isolated cases (82.2% vs. 48.6%) (*p* < 0.001). Among patients with LR, high grade (OR 2.79, 95% CI 1.42–5.49) and axial location (OR 2.28, 95% CI 1.17–4.47) were independently associated with combined recurrence. In the combined phenotype, metastasis preceded LR in 40.0%, LR preceded metastasis in 34.3%, and events were synchronous in 25.7%. **Conclusions**: Recurrence in STS comprises distinct phenotypes defined by the interaction of local and metastatic disease. In particular, the clinical significance of local recurrence depends on its metastatic context.

## Simple Summary

Soft tissue sarcomas are rare cancers that may recur locally, metastasize, or both. These events are usually analyzed separately, which may obscure clinically relevant patterns of disease progression. In this study, we analyzed 668 patients and identified four recurrence phenotypes: no event (52.2%), metastasis only (23.8%), local recurrence only (13.5%), and combined local recurrence and metastasis (10.5%). Local recurrence did not behave as a uniform clinical pattern. When it occurred together with metastasis, it was associated with more aggressive tumor features and earlier recurrence, whereas isolated local recurrence occurred later and appeared more closely linked to local treatment factors. These findings suggest that recurrence in soft tissue sarcoma is better understood as distinct phenotypes rather than a single endpoint, which may improve the interpretation of recurrence patterns, outcome analyses, and future risk-adapted disease modeling.

## 1. Introduction

Soft tissue sarcomas (STS) comprise a heterogeneous group of mesenchymal malignancies with diverse biological behavior and clinical outcomes [1,2,3]. Despite advances in multidisciplinary management, disease progression remains a major clinical challenge, with local recurrence (LR) and distant metastasis representing the principal determinants of long-term outcome [4,5,6].

Traditionally, LR and metastasis have been analyzed as separate clinical endpoints. However, this endpoint-based approach may obscure relevant patterns of disease evolution. In particular, LR is often treated as a uniform event, although its prognostic significance appears to vary substantially across clinical contexts [7,8,9,10]. While some patients develop isolated local failure with prolonged disease control, others experience LR in parallel with aggressive metastatic progression. This suggests that LR may not represent a single biological entity, but rather different clinical states whose meaning depends on their metastatic context [11].

The relationship between LR and metastasis remains incompletely understood. Prior studies have reported inconsistent findings regarding the prognostic impact of LR, with some demonstrating an association with survival and others not. One possible explanation is that conventional analyses group biologically distinct recurrence trajectories into overly broad endpoint categories. If so, recurrence in STS may be more appropriately interpreted as a structured phenotype architecture rather than as isolated binary events [12,13,14,15,16].

In addition, the temporal dynamics of recurrence may provide further insight into tumor biology. Early recurrence has been associated with more aggressive disease behavior, whereas late recurrence may reflect more localized or treatment-related processes. Likewise, the sequence of LR and metastatic progression may help distinguish parallel disease processes from simpler linear progression models [17,18,19,20,21].

Therefore, the aim of this study was to characterize recurrence in STS as a phenotype architecture defined by the interaction between local and metastatic disease. By distinguishing patients with no event, isolated metastasis, isolated local recurrence, and combined recurrence, and by analyzing the timing and sequence of events, we sought to determine whether these patterns represent clinically and biologically distinct disease trajectories.

## 2. Materials and Methods

### 2.1. Study Design and Swiss Sarcoma Network

Patients were identified through the Swiss Sarcoma Network (SSN) registry (SSN-ShapeHub^®^-V1), a nationwide prospective database initiated in 2018 that captures clinical, pathological, treatment, and follow-up information for patients discussed within the network [22]. Clinical information is recorded following review at the weekly multidisciplinary sarcoma board (MDT/SB), allowing harmonized documentation of diagnostic findings, treatment decisions, and follow-up events. These meetings ensure continuous data validation, harmonized documentation, and transdisciplinary decision-making. Data captured within the SSN include diagnostic workup, tumor characteristics, treatment strategies, surgical outcomes, and follow-up events, providing a high-quality dataset for evaluating disease trajectories in sarcoma patients.

### 2.2. Study Population and Data Extraction

**Figure 1:**
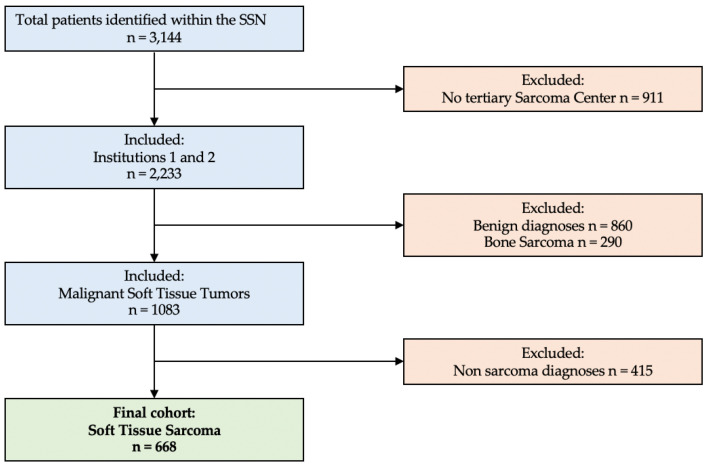
Flowchart of patient selection. From 3144 patients identified in the Swiss Sarcoma Network, 911 not treated at tertiary centers were excluded, leaving 2233 patients at Institution 1 and Institution 2. After exclusion of benign diagnoses (*n* = 860) and bone sarcomas (*n* = 290), 1083 malignant soft tissue tumors remained. Following removal of non-sarcoma diagnoses (*n* = 415), the final cohort comprised 668 patients with histologically confirmed soft tissue sarcoma.

All consecutive patients presented to the SSN MDT/SB between 2018 and 2025 were eligible for inclusion, irrespective of prior treatment status, recurrence status, or disease extent (localized or metastatic disease), thereby reflecting a real-world sarcoma population. For the present analysis, a stepwise selection process was applied. First, only patients treated at two tertiary sarcoma referral centers (Institution 1 and Institution 2) were included to ensure data completeness and comparability. Second, patients with primary bone sarcomas were excluded. Third, patients with STS located in extremities, trunk, head and neck, or visceral/retroperitoneal sites were retained. Finally, lesions not classified as STS according to the WHO Classification of Soft Tissue and Bone Tumours were excluded [3]. This included non-neoplastic lesions, tumor simulators, and selected mesenchymal or hematologic entities not considered soft tissue sarcomas within the WHO framework or not applicable to conventional AJCC soft tissue sarcoma staging [23] (Figure 1).

After application of all criteria, the final cohort comprised 668 patients with histologically confirmed soft tissue sarcoma. Data were extracted using the Adjumed platform (Adjumed Services AG, Zurich, Switzerland; accessed December 2025), including demographic variables, tumor characteristics, treatment data, and outcomes.

### 2.3. Data Integration and Quality Control

A structured quality control framework was applied to ensure consistency between the main clinical dataset and the metastatic dataset. Automated checks identified chronological inconsistencies, missing key event information, and conflicting entries across sources. Discrepant cases were reviewed using predefined prioritization rules and cross-checked against MDT/SB documentation where available. Records with unresolved inconsistencies were manually reviewed and excluded from specific analyses if necessary.

### 2.4. Definitions, Clinical Variables, and Outcome Measures

Tumor classification was performed by reference sarcoma pathologists according to contemporary WHO criteria, using morphology, immunohistochemistry, and molecular testing where appropriate. All cases were discussed within the multidisciplinary sarcoma board.

Tumor-related variables included anatomical site (extremity, axial, intra-abdominal/retroperitoneal, head and neck, urogenital/perineal/anal), tumor depth (superficial vs. deep), tumor grade (G1–G3), and tumor class (malignant vs. intermediate). Resection margin status was categorized as R0, R1, or R2 in patients undergoing surgical resection [24,25,26,27]. Patients without surgical treatment were classified as “no surgery,” and cases with unavailable information were categorized as “not applicable/missing.”

Patients were stratified into four predefined recurrence phenotypes: no event, metastasis only, local recurrence only, and combined local recurrence and metastatic disease. Metastatic disease included both synchronous metastatic disease present at initial diagnosis (M1) and metachronous metastatic progression occurring during follow-up.

Baseline staging generally included cross-sectional chest imaging according to institutional sarcoma protocols; however, the present registry analysis relied on documented metastatic status and event dates rather than central radiological re-review of all baseline imaging. Patients with primary metastatic disease were retained because the aim of the analysis was to characterize overall patterns of local and systemic disease progression rather than exclusively recurrence after curative-intent treatment.

Time zero was defined as the date of definitive surgical treatment in patients undergoing resection. In patients without surgery, the date of histological diagnosis was used as the reference time point. LR was defined as tumor reappearance at or adjacent to the primary tumor site following prior local treatment. Patients without surgical treatment were therefore not classified as having LR.

LR was further categorized according to timing. Early local recurrence was defined as the occurrence within 365 days after the reference time point, whereas late local recurrence was defined as the occurrence beyond 365 days [11].

Patients undergoing unplanned (“whoops”) resections were considered surgically treated and were therefore eligible for classification as local recurrence in the event of subsequent tumor reappearance.

In patients who developed both local recurrence and metastatic disease, the temporal sequence of events was additionally assessed. Event sequences were classified as metastasis preceding local recurrence, local recurrence preceding metastasis, or synchronous occurrence. Synchronous disease was defined as both events occurring within 30 days without a clearly distinguishable temporal order.

### 2.5. Statistical Analysis

Descriptive statistics were used to summarize patient demographics, tumor characteristics, treatment variables, and outcomes. Categorical variables are presented as counts and percentages, and continuous variables as mean ± standard deviation.

Comparisons across recurrence phenotypes were performed using chi-square or Fisher’s exact tests for categorical variables and one-way ANOVA or Kruskal–Wallis tests for continuous variables, as appropriate. *p*-values are reported for descriptive purposes and were not adjusted for multiple comparisons.

Recurrence phenotype distribution and event sequences were analyzed descriptively and visualized using bar charts and Sankey diagrams.

To assess differences between isolated local recurrence and combined recurrence, a multivariable logistic regression model was performed, restricted to patients with local recurrence. The outcome was combined disease (yes/no). Covariates (tumor grade, tumor depth, anatomical location, and resection margin status) were selected a priori based on clinical relevance. Results are reported as odds ratios (ORs) with 95% confidence intervals (CIs).

Because atypical lipomatous tumor/well-differentiated liposarcoma (ALT/WDLPS) represents a biologically context-dependent entity with limited metastatic potential in peripheral locations, a predefined sensitivity analysis was performed to evaluate the robustness of the phenotype framework. Peripheral and superficial ALT/WDLPS cases were excluded, whereas retroperitoneal and intra-abdominal WDLPS were retained in accordance with WHO classification principles due to their distinct biological behavior and potential for dedifferentiation. Following exclusion, recurrence phenotype distribution and key biological associations were reassessed descriptively.

Given the observational design and outcome-defined phenotype groups, treatment variables were interpreted as descriptive and hypothesis-generating rather than causal. Statistical analyses were conducted using SAS software version 9.4 (SAS Institute, Cary, NC, USA), and a two-sided *p*-value < 0.05 was considered indicative of statistical significance.

## 3. Results

### 3.1. Study Patient Population

Patients were initially identified from the SSN database (*n* = 3144). In a first step, patients not treated at tertiary sarcoma referral centers were excluded (*n* = 911), and the analysis was restricted to cases managed at Institutions 1 and 2 (*n* = 2233). This restriction ensured a homogeneous, high-quality dataset and enabled consistent comparison between specialized centers. In the second step, benign diagnoses (*n* = 860) and primary bone sarcomas (*n* = 290) were excluded, resulting in a cohort of patients with malignant soft tissue tumors (*n* = 1083). In the final step, lesions not classified as STS according to the WHO Classification of Soft Tissue and Bone Tumours were excluded (*n* = 415). These included entities not considered soft tissue sarcomas within the WHO framework or not applicable to conventional AJCC soft tissue sarcoma staging. The final study cohort consisted of 668 patients with histologically confirmed soft tissue sarcoma (Figure 1).

#### Distribution of Excluded and Included Entities

**Figure 2:**
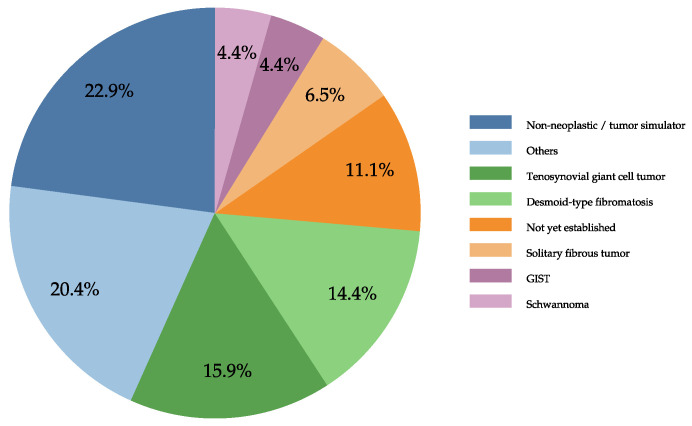
Distribution of excluded non-sarcoma entities. The pie chart shows the distribution of diagnostic categories among excluded cases. Non-neoplastic lesions/tumor simulators (22.9%) and “others” (20.4%) were most common, followed by tenosynovial giant cell tumor (15.9%) and desmoid-type fibromatosis (14.4%). Additional categories included not yet established (11.1%), solitary fibrous tumor (6.5%), GIST (4.4%), and schwannoma (4.4%).

**Figure 3:**
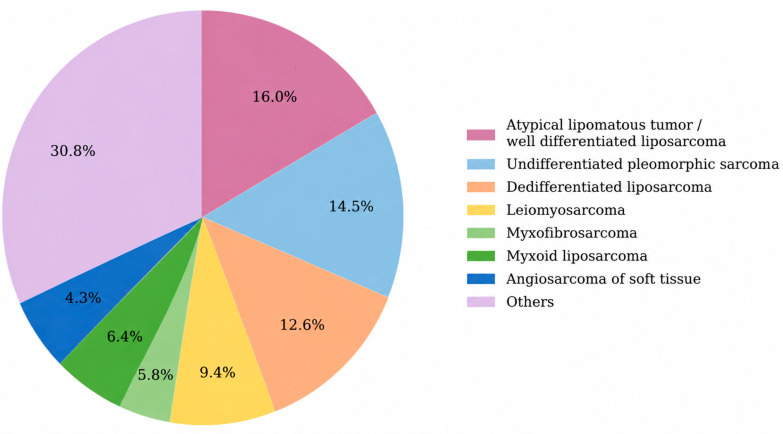
Distribution of included sarcoma subtypes. The pie chart shows the distribution of histological subtypes in the study cohort. Atypical lipomatous tumor/well-differentiated liposarcoma was the most frequent entity (16.0%), followed by undifferentiated/unclassified sarcoma (14.5%), dedifferentiated liposarcoma (12.6%), leiomyosarcoma (9.4%), myxoid liposarcoma (6.4%), myxofibrosarcoma (5.8%), and angiosarcoma of soft tissue (4.3%). The remaining histological subtypes, each accounting for less than 4%, were grouped as “others” (30.8%).

The distribution of excluded non-sarcoma entities is shown in Figure 2, with only diagnoses accounting for more than 4% of cases presented individually and less frequent entities grouped as “others.” The distribution of histological subtypes within the cohort is summarized in Figure 3. Atypical lipomatous tumor/well-differentiated liposarcoma was the most frequent subtype, followed by undifferentiated/unclassified sarcoma, dedifferentiated liposarcoma, and leiomyosarcoma, while less common subtypes were grouped as “others.” Overall, the distribution reflects typical patterns observed in adult soft tissue sarcoma cohorts.

Additional analysis of ALT/WDLPS according to anatomical location demonstrated that 15 of 107 cases (14.0%) were located intra- or retroperitoneally, whereas the majority occurred in the extremities. This distinction was considered relevant given the different biological behavior of retroperitoneal versus peripheral ALT/WDLPS.

### 3.2. Baseline Characteristics Across Recurrence Phenotypes (Table 1)

**Figure 4:**
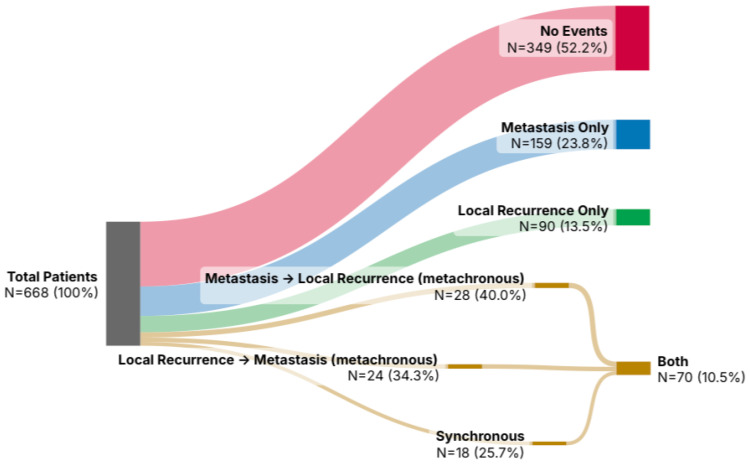
Sankey diagram of recurrence phenotypes and event sequences. The diagram illustrates the distribution of recurrence phenotypes in the cohort (*n* = 668) and the temporal sequence of events among patients with combined local recurrence and metastatic disease (*n* = 70). Phenotypes were distributed as no event (52.2%), metastasis only (23.8%), local recurrence only (13.5%), and combined disease (10.5%). Within the combined phenotype, event sequences were categorized into three mutually exclusive groups: metastasis preceding local recurrence (40.0%), local recurrence preceding metastasis (34.3%), and synchronous occurrence of both events (25.7%). Together, these categories accounted for all patients in the combined phenotype, without a dominant sequence pattern.

A total of 668 patients with histologically confirmed STS were included in the analysis. Patients were stratified into four phenotype groups: no event (*n* = 349, 52.2%), metastasis only (*n* = 159, 23.8%), local recurrence only (*n* = 90, 13.5%), and both local recurrence and metastasis (*n* = 70, 10.5%). The relationship between recurrence phenotypes and the temporal sequence of events is illustrated in Figure 4.

**Table 1:** Baseline characteristics of the study cohort stratified by recurrence phenotype. The values are presented as numbers (percentages) for categorical variables and as mean ± standard deviation for continuous variables.

Variable	Overall (*n* = 668, 100%)	No Event (*n* = 349, 52.2%)	Met Only (*n* = 159, 23.8%)	LR Only (*n* = 90, 13.5%)	Both (*n* = 70, 10.5%)	*p*-Value
**Patient characteristics**						
**Sex**						0.185
Male	346 (51.8%)	190 (54.5%)	71 (44.7%)	50 (55.6%)	35 (50.0%)	
Female	322 (48.2%)	159 (45.6%)	88 (55.3%)	40 (44.4%)	35 (50.0%)	
**Institution**						0.721
Institution 1	384 (57.5%)	197 (56.4%)	97 (61.0%)	49 (54.4%)	41 (58.6%)	
Institution 2	284 (42.5%)	152 (43.6%)	62 (39.0%)	41 (46.6%)	29 (41.4%)	
**Age (years)**	59.8 ± 17.2	60.1 ± 17.4	59.6 ± 17.8	60.0 ± 16.6	58.0 ± 16.1	0.810
**Tumor characteristics**						
**Depth**						<0.001
Deep	555 (83.1%)	268 (76.8%)	147 (92.5%)	76 (84.4%)	64 (91.4%)	
Superficial	113 (16.9%)	81 (23.2%)	12 (7.5%)	14 (15.9%)	6 (8.3%)	
**Region**						<0.001
Lower extremity	253 (37.9%)	152 (43.6%)	53 (33.3%)	32 (35.6%)	16 (22.9%)	
Face, head and neck	34 (5.1%)	17 (4.9%)	11 (6.9%)	2 (2.2%)	4 (5.7%)	
Intra- and retroperitoneal	145 (21.7%)	54 (15.5%)	44 (27.7%)	23 (25.6%)	24 (34.3%)	
Axial	83 (12.4%)	49 (14.0%)	13 (8.2%)	13 (14.4%)	8 (11.4%)	
Upper extremity	91 (13.6%)	51 (14.6%)	16 (10.1%)	15 (17.7%)	9 (12.9%)	
Urogenital, perianal and anal	58 (8.7%)	25 (7.2%)	19 (11.9%)	5 (5.6%)	9 (12.9%)	
n/a	4 (0.6%)	1 (0.3%)	3 (1.9%)	0 (0.0%)	0 (0.0%)	
**Grading**						<0.001
G1	223 (33.4%)	169 (48.4%)	11 (6.9%)	34 (37.8%)	9 (12.9%)	
G2	145 (21.7%)	79 (22.6%)	31 (19.5%)	21 (23.3%)	14 (20.0%)	
G3	298 (44.6%)	101 (28.9%)	116 (73.0%)	35 (38.9%)	46 (65.7%)	
n/a	2 (0.3%)	0 (0.0%)	1 (0.6%)	0 (0.0%)	1 (1.4%)	
**R-Status**						<0.001
R0	376 (56.3%)	239 (68.5%)	81 (50.9%)	28 (31.1%)	28 (40.0%)	
R1	131 (19.6%)	57 (16.3%)	17 (10.7%)	33 (36.7%)	24 (34.3%)	
R2	29 (4.3%)	4 (1.1%)	10 (6.3%)	7 (7.8%)	8 (11.4%)	
No surgery	81 (12.1%)	42 (12.0%)	39 (24.5%)	0 (0.0%)	0 (0.0%)	
n/a	51 (7.6%)	7 (2.0%)	12 (7.5%)	22 (24.4%)	10 (14.3%)	
**Treatment**						
**Whoops**						0.033
No	486 (72.8%)	255 (73.1%)	127 (79.9%)	55 (61.1%)	49 (70.0%)	
Yes	176 (26.3%)	93 (26.6%)	30 (18.9%)	33 (36.7%)	20 (28.6%)	
n/a	6 (0.9%)	1 (0.3%)	2 (1.2%)	2 (2.2%)	1 (1.4%)	
**Radiotherapy**						<0.001
No	329 (49.3%)	212 (60.7%)	53 (33.3%)	48 (53.3%)	16 (22.9%)	
Yes	339 (50.7%)	137 (39.3%)	106 (66.7%)	42 (46.7%)	54 (77.1%)	
**Chemotherapy**						<0.001
No	501 (75.0%)	328 (94.0%)	72 (45.3%)	79 (87.8%)	22 (31.4%)	
Yes	167 (25.0%)	21 (6.0%)	87 (54.7%)	11 (12.2%)	48 (68.6%)	
**Treatment overlap**						<0.001
Chemo only	44 (6.6%)	9 (2.6%)	21 (13.2%)	5 (5.6%)	9 (12.9%)	
RT only	216 (32.3%)	125 (35.8%)	40 (25.2%)	36 (40.0%)	15 (21.4%)	
RT and Chemo	123 (18.4%)	12 (3.4%)	66 (41.5%)	6 (6.7%)	39 (55.7%)	
Neither	285 (42.7%)	203 (58.2%)	32 (20.1%)	43 (47.8%)	7 (10.0%)	
**Outcome**						
**Local recurrence**						
No	508 (76.0%)	349 (100.0%)	159 (100.0%)	0 (0.0%)	0 (0.0%)	
Yes	160 (24.0%)	0 (0.0%)	0 (0.0%)	90 (100.0%)	70 (100.0%)	
**Metastasis**						
No	439 (65.4%)	349 (100.0%)	0 (0.0%)	90 (100.0%)	0 (0.0%)	
Yes	229 (34.6%)	0 (0.0%)	159 (100.0%)	0 (0.0%)	70 (100.0%)	
**Early vs. late local recurrence**						<0.001
Early LR	52 (32.5%)	0 (0.0%)	0 (0.0%)	16 (17.8%)	36 (51.4%)	
Late LR	108 (67.5%)	0 (0.0%)	0 (0.0%)	74 (82.2%)	34 (48.6%)	

*p*-values were calculated using chi-square tests for categorical variables and one-way analysis of variance (ANOVA) for continuous variables. Fisher’s exact test was applied when expected cell counts were low. *p*-values are reported for descriptive and exploratory purposes and were not adjusted for multiple comparisons; therefore, they should be interpreted cautiously and not as confirmatory evidence of independent associations. Variables defining the recurrence phenotypes, including local recurrence and metastatic status, are presented for completeness but are not interpreted inferentially, as differences between groups are inherent to the study design. Similarly, variables reflecting downstream clinical events (e.g., early versus late local recurrence) are descriptive and should not be interpreted as baseline predictors. Resection margin status was categorized as R0, R1, or R2 in patients undergoing surgical resection. Patients without surgical treatment were classified as “no surgery,” and cases with unavailable information were categorized as “not applicable.” Unplanned (“whoops”) resections were defined as surgical excisions performed without prior oncologic planning or appropriate preoperative workup. Abbreviations: LR, local recurrence; Met, metastasis; R0, microscopically negative margin; R1, microscopically positive margin; R2, macroscopically positive margin; RT, radiotherapy; CT, chemotherapy; n/a, not available.

Follow-up duration differed across recurrence phenotypes. Median follow-up for the overall cohort was 27.6 months (IQR 9.7–58.3 months). Patients without events and those with isolated local recurrence demonstrated the longest follow-up durations, with median follow-up times of 39.8 months (IQR 20.4–79.1) and 46.7 months (IQR 24.3–88.5), respectively. In contrast, patients with metastasis-only disease had the shortest follow-up (18.6 months, IQR 7.9–39.2), whereas patients with combined local recurrence and metastatic disease showed intermediate follow-up durations (28.1 months, IQR 13.5–56.4). These differences are consistent with the distinct clinical trajectories observed across recurrence phenotypes.

#### 3.2.1. Patient Characteristics

Baseline demographic variables did not meaningfully differ across phenotype groups. The proportion of male patients ranged from 44.7% in the metastasis-only group to 55.6% in the local recurrence-only group (*p* = 0.185). Mean age at diagnosis was comparable across groups (59.8 ± 17.2 years overall; *p* = 0.810), and no relevant differences were observed between treating institutions (*p* = 0.721). These findings indicate that patient-related factors do not account for the observed phenotype structure.

#### 3.2.2. Tumor Characteristics

In contrast, tumor-related variables showed marked and consistent differences across phenotype groups. High-grade tumors (G3) were strongly enriched in phenotypes involving metastatic disease, accounting for 73.0% of cases in the metastasis-only group and 65.7% in the combined phenotype, compared to 28.9% in patients without events and 38.9% in those with isolated LR (*p* < 0.001). Conversely, low-grade tumors (G1) were most frequent in the no-event group (48.4%) and markedly underrepresented in metastasis-only disease (6.9%).

Tumor depth also differed significantly, with deep tumors present in 92.5% of metastasis-only cases and 91.4% of combined cases, compared to 76.8% in the no-event group (*p* < 0.001). Similarly, tumor location varied across phenotypes, with intra-abdominal and retroperitoneal tumors more frequently observed in patients with combined recurrence (34.3%) compared to those without events (15.5%) (*p* < 0.001).

#### 3.2.3. Treatment Characteristics

Treatment-related variables differed significantly across phenotype groups but should be interpreted as descriptive, reflecting clinical decision-making rather than causal effects.

Radiotherapy was more frequently administered in patients with combined recurrence (77.1%) and metastasis-only disease (66.7%) compared with patients without events (39.3%) (*p* < 0.001). Similarly, chemotherapy use was highest in the combined phenotype (68.6%) and lowest in patients without events (6.0%) (*p* < 0.001).

Unplanned (“whoops”) resections showed a distinct pattern across phenotypes (*p* = 0.033). The highest proportion was observed in patients with isolated LR (36.7%), followed by those with combined recurrence (28.6%), compared to 26.6% in patients without events and 18.9% in the metastasis-only group. This distribution is compatible with a stronger contribution of local management factors in isolated LR, although causal inference cannot be established.

#### 3.2.4. Surgical Factors

Resection margin status differed significantly across phenotype groups (*p* < 0.001) and showed one of the clearest phenotype-specific patterns.

Complete resections (R0) were most frequent in patients without events (68.5%), compared to 50.9% in the metastasis-only group, 40.0% in the combined phenotype, and 31.1% in patients with isolated LR. In contrast, incomplete resections (R1/R2) were more common in LR phenotypes, particularly in the combined group (34.3% R1).

Notably, 24.5% of patients in the metastasis-only group did not undergo surgical resection and were classified as “no surgery,” highlighting fundamental differences in treatment pathways between phenotypes. These findings suggest that isolated LR is more closely associated with local control factors, whereas metastatic phenotypes are influenced by both biological aggressiveness and treatment context.

#### 3.2.5. Outcome Patterns and Recurrence Timing

By definition, the occurrence of LR and metastasis differed across phenotype groups. Importantly, the timing of LR provided additional discriminatory information.

Among patients with LR, early recurrence (≤365 days) was substantially more frequent in the combined phenotype (51.4%) compared to isolated local recurrence (17.8%), whereas late recurrence (>365 days) predominated in isolated local recurrence (82.2%) compared to the combined phenotype (48.6%) (*p* < 0.001). These findings indicate that early recurrence is strongly associated with metastatic disease, whereas late recurrence is more often confined to the local site.

Overall, the combined phenotype was characterized by high-grade, deep-seated tumors and a higher proportion of early recurrence, whereas isolated local recurrence exhibited a comparatively less aggressive tumor profile and a predominance of late recurrence. This pattern supports the concept of biologically distinct disease trajectories.

To evaluate the potential impact of follow-up duration on phenotype assignment, a sensitivity analysis restricted to patients with at least 24 months of follow-up was performed (*n* = 357). Although the relative distribution of phenotypes changed modestly, the overall phenotype architecture remained stable. Phenotypes involving metastatic disease continued to demonstrate enrichment for high-grade tumors, whereas isolated local recurrence retained a comparatively less aggressive profile. Early local recurrence remained more frequent in the combined phenotype than in isolated local recurrence, supporting the robustness of the observed recurrence patterns despite longer observation requirements.

The distribution of recurrence phenotypes varied across histological subtypes, although these findings should be interpreted descriptively because several subgroups contained limited numbers of patients. Detailed histology-specific phenotype distributions are presented in Appendix A, Table A1.

### 3.3. Local Recurrence–Specific Heterogeneity

**Figure 5:**
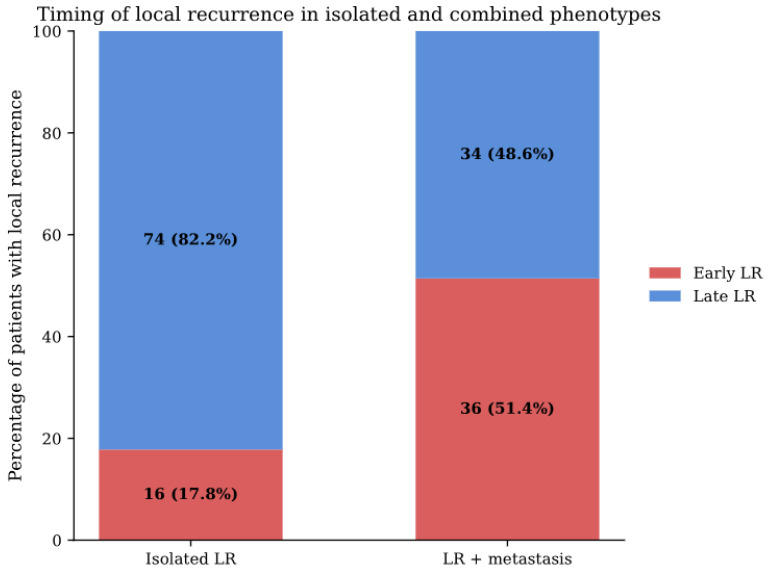
Distribution of early local recurrence (≤365 days) and late local recurrence (>365 days) among patients with isolated local recurrence and those with combined local recurrence and metastatic disease. Early recurrence was substantially more frequent in the combined phenotype (51.4%) compared to isolated local recurrence (17.8%), whereas late recurrence predominated in isolated local recurrence (82.2% vs. 48.6%).

To directly assess the heterogeneity of local recurrence (LR), patients with isolated LR were compared to those with combined LR and metastatic disease. Patients with combined recurrence exhibited a higher proportion of high-grade tumors (65.7% vs. 38.9%) and deep tumor location (91.4% vs. 84.4%) compared to those with isolated LR. Most notably, recurrence timing differed substantially between groups. Early local recurrence occurred in 51.4% of patients with combined disease compared to 17.8% in isolated LR, whereas late recurrence predominated in isolated cases (82.2% vs. 48.6%) (Figure 5).

These findings demonstrate that LR in STS does not represent a single clinical entity. Rather, isolated LR and LR occurring in the context of metastatic disease represent phenotypically distinct trajectories with different biological signatures and temporal dynamics. Together, these results indicate that the clinical significance of local recurrence is not uniform but critically depends on its metastatic context.

Sensitivity analysis excluding peripheral/superficial ALT/WDLPS demonstrated a stable overall phenotype architecture. Metastatic phenotypes remained strongly enriched for high-grade tumors, whereas isolated LR retained a comparatively more intermediate biological profile. These findings indicate that the observed phenotype patterns were not solely driven by the inclusion of intermediate locally aggressive entities.

### 3.4. Multivariable Analysis of Local Recurrence Phenotypes

To evaluate whether these differences persisted after adjustment for tumor characteristics, a multivariable logistic regression analysis was performed, restricted to patients with LR.

High tumor grade (G3 vs. G1–2) was independently associated with the development of combined LR and metastatic disease (OR 2.79, 95% CI 1.42–5.49, *p* = 0.003). Axial tumor location was also independently associated with combined disease (OR 2.28, 95% CI 1.17–4.47, *p* = 0.016). In contrast, tumor depth (OR 2.14, 95% CI 0.73–6.25, *p* = 0.163) and resection margin status (OR 0.93, 95% CI 0.45–1.92, *p* = 0.839) were not independently associated (Table 2).

These findings indicate that the distinction between isolated LR and combined recurrence is not solely explained by local control factors but is strongly influenced by underlying tumor biology.

### 3.5. Sequence of Events in the Combined Phenotype

Among patients with both LR and metastatic disease (*n* = 70), the temporal sequence of events was heterogeneous. Metastasis preceded local recurrence in 28 patients (40.0%), whereas local recurrence occurred first in 24 patients (34.3%). In 18 patients (25.7%), both events occurred within a similar time frame and were classified as synchronous (Table 3). These categories were mutually exclusive and together accounted for all patients in the combined phenotype.

The absence of a dominant sequence pattern indicates that no single progression pathway explains the combined phenotype. Instead, these findings support a model of parallel or partially independent disease processes, consistent with biologically heterogeneous trajectories in soft tissue sarcoma.

## 4. Discussion

### 4.1. Main Findings

This study demonstrates that recurrence in STS is not adequately described by isolated endpoints but is better understood as a phenotype architecture defined by the interaction between LR and metastatic disease. Patients with isolated LR and those with LR in the context of metastasis represent distinct clinical trajectories rather than variations of a single recurrence process. The distribution of recurrence phenotypes and the consistent differences observed across tumor characteristics, timing, and event sequence support the existence of biologically and clinically meaningful subgroups [28,29,30].

A central implication of these findings is that LR in STS cannot be interpreted independently of metastatic context. Isolated LR and LR occurring in conjunction with metastatic disease do not represent different severities of a single endpoint, but rather distinct clinical phenotypes with different biological signatures and temporal dynamics. This distinction provides a coherent explanation for the inconsistent prognostic significance of LR reported in prior studies, as analyses that treat LR as a homogeneous event may inadvertently combine biologically divergent patient groups. In this sense, the present data support a shift from endpoint-based to phenotype-based interpretation of recurrence.

### 4.2. Biological Interpretation of Recurrence Phenotypes

The observed differences between recurrence phenotypes were primarily driven by tumor-related characteristics rather than patient demographics, supporting a biological basis for phenotype differentiation. High-grade tumors accounted for 73.0% of metastasis-only cases and 65.7% of combined recurrence, compared to 38.9% in isolated LR and 28.9% in patients without events. Similarly, deep tumor location and malignant histology were strongly enriched in phenotypes involving metastatic disease. These findings are consistent with established determinants of sarcoma aggressiveness and suggest that the combined phenotype reflects tumors with higher intrinsic metastatic potential [14,31,32,33].

The timing of LR further reinforces this distinction. Early recurrence (≤365 days) occurred in 51.4% of patients with combined recurrence compared to 17.8% in isolated LR, whereas late recurrence predominated in isolated cases (82.2% vs. 48.6%). This pattern indicates that early LR is more closely associated with aggressive, systemically active disease, whereas late LR is more often confined to the local compartment. In addition, the absence of a dominant event sequence—metastasis preceding LR in 40.0%, LR preceding metastasis in 34.3%, and synchronous occurrence in 25.7%—argues against a unidirectional progression model. Instead, these findings support a framework of parallel or partially independent disease processes driven by underlying tumor biology [8,11,17].

Because inclusion of ALT/WDLPS may introduce biological heterogeneity depending on anatomical location, additional sensitivity analyses excluding peripheral/superficial ALT/WDLPS were performed. The overall phenotype architecture and the association between metastatic phenotypes and aggressive tumor characteristics remained stable, supporting the robustness of the observed recurrence patterns.

Similarly, sensitivity analysis restricted to patients with at least 24 months of follow-up demonstrated stable phenotype patterns, suggesting that the principal findings were not solely driven by limited observation time.

### 4.3. Local Recurrence as a Heterogeneous Entity

Building on this biological framework, LR itself emerges as a heterogeneous entity rather than a uniform clinical endpoint. Traditionally, LR has been treated as a single endpoint and often interpreted as a surrogate of aggressive disease. The present findings challenge this assumption. By separating isolated LR from LR occurring in the context of metastatic disease, we show that these represent phenotypically distinct conditions with different biological signatures and temporal dynamics. This distinction provides a direct explanation for the long-standing inconsistency in the literature regarding the prognostic impact of LR, as studies that do not differentiate between isolated and metastasis-associated LR effectively analyze biologically distinct patient populations as a single group [7,32,34,35,36].

Importantly, LR cannot be interpreted exclusively as a manifestation of tumor biology. Treatment-related and system-level factors also appear to contribute. The higher proportion of unplanned (“whoops”) resections in isolated LR (36.7%) compared to metastasis-only disease (18.9%) suggests that local management factors may play a more prominent role in certain recurrence phenotypes. Similarly, the strong association between resection margin status and LR supports the contribution of local control. These findings indicate that recurrence phenotypes reflect an interplay between tumor biology and treatment quality rather than a single underlying mechanism [37,38,39,40,41]. More broadly, these findings suggest that conventional endpoint- or risk-based frameworks may remain biologically too coarse, as they aggregate patients with fundamentally different patterns of local and systemic disease progression.

### 4.4. Implications for Current Concepts of Local Recurrence

These results have implications for how recurrence is conceptualized in STS. Current frameworks typically treat LR and metastasis as separate endpoints or combine them into composite outcomes, which may obscure clinically relevant heterogeneity. The phenotype-based approach presented here suggests that recurrence should instead be interpreted within a multidimensional framework that accounts for the interaction between local and systemic disease [6,33,42,43,44].

This perspective may also explain inconsistencies in the literature regarding the prognostic impact of LR. Studies that treat LR as a homogeneous event may fail to detect meaningful associations because they aggregate distinct phenotypes with different biological behaviors. A phenotype-based framework may therefore improve both the interpretability of recurrence outcomes and the development of more refined prognostic models [45,46,47,48,49].

### 4.5. Clinical Implications

Although this study was not designed to guide treatment decisions, several clinically relevant implications emerge. First, the observed differences between recurrence phenotypes suggest that local and systemic disease progression in STS may not represent a uniform process. Patients with combined recurrence exhibited earlier events and more aggressive tumor characteristics, whereas isolated local recurrence was associated with longer follow-up durations and a predominance of late recurrence, supporting the concept of a more locally confined disease trajectory. These findings may inform future studies investigating risk-adapted surveillance approaches, although the present analysis does not establish that intensified surveillance improves outcomes in any recurrence phenotype.

Second, the findings highlight the importance of optimal initial management. The association between whoops resections, margin status, and local recurrence suggests that a subset of recurrence events may be influenced by modifiable treatment factors. While causality cannot be established, these patterns reinforce the importance of early referral and multidisciplinary care in specialized sarcoma centers.

Finally, incorporating recurrence phenotypes into prognostic frameworks may provide a more nuanced understanding of disease progression than models based solely on baseline tumor characteristics.

### 4.6. Relation to Existing Literature

STS is widely recognized as a heterogeneous disease group with diverse histological subtypes and clinical behaviors [3]. Previous studies have examined risk factors for LR and metastasis individually, but few have explored their interaction in a structured manner. Some reports have suggested an association between LR and metastatic risk, although the direction and causality of this relationship remain unclear [8,50,51,52].

The present study extends this literature by demonstrating that LR is not a uniform event and that its clinical significance depends on its relationship with metastatic disease. By integrating LR and metastasis into a unified phenotype framework, this work provides a conceptual explanation for previously inconsistent findings and offers a more structured approach to interpreting recurrence patterns.

### 4.7. Future Directions

The phenotype-based framework proposed in this study provides a foundation for further investigation of recurrence dynamics in STS. Future studies should explore the prognostic relevance of recurrence timing and sequence, particularly with respect to the temporal relationship between LR and metastatic progression. In addition, distinguishing recurrence driven by aggressive tumor biology from recurrence related to local treatment factors remains a critical challenge with direct clinical implications. More advanced analytical approaches, including multi-state modeling of disease transitions, may further refine the understanding of sarcoma progression. Finally, histology-specific analyses will be essential to determine whether the relationship between local and systemic recurrence differs across sarcoma subtypes.

## 5. Limitations

Several limitations should be acknowledged. First, the retrospective nature of the analysis limits causal inference. Second, recurrence phenotypes were defined based on observed outcomes and therefore cannot be directly applied for baseline risk prediction. Third, treatment-related variables are subject to confounding by indication and should be interpreted descriptively rather than causally. Fourth, the analysis was restricted to two tertiary sarcoma centers to ensure data quality and comparability, which may limit generalizability. In addition, inclusion of intermediate locally aggressive entities, particularly ALT/WDLPS, may introduce biological heterogeneity because metastatic potential depends strongly on anatomical context. However, sensitivity analyses excluding peripheral/superficial ALT/WDLPS demonstrated stable phenotype patterns. Furthermore, operational definitions of recurrence timing and synchronous disease were based on registry-derived event dates and may have been influenced by differences in imaging intervals and follow-up schedules. Furthermore, recurrence phenotypes were assigned based on observed events during follow-up. Consequently, some patients classified as isolated local recurrence or metastasis-only disease may transition to combined recurrence with longer observation. Although sensitivity analysis restricted to patients with at least 24 months of follow-up demonstrated stable phenotype patterns, longer-term follow-up and time-dependent analytical approaches are needed to further evaluate phenotype stability over time. In addition, death may act as a competing event for subsequent local recurrence, particularly among patients with aggressive metastatic disease. This may lead to underestimation of local recurrence in rapidly progressive phenotypes and should be considered when interpreting phenotype distributions. Finally, external validation in independent cohorts is required to confirm the reproducibility of the proposed phenotype framework.

## 6. Conclusions

Recurrence in soft tissue sarcoma is not a uniform process but comprises distinct phenotypes defined by the interaction between local recurrence and metastatic disease. In particular, the clinical significance of local recurrence depends on its metastatic context: isolated local recurrence differs fundamentally from recurrence occurring in conjunction with metastasis and reflects a different biological and clinical trajectory. These findings support a shift from endpoint-based to phenotype-based interpretation of recurrence and provide a framework for more refined analysis of disease progression in soft tissue sarcoma.

## Figures and Tables

**Table 2:** Multivariable logistic regression analysis of factors associated with combined local recurrence and metastatic disease compared to isolated local recurrence.

Variable	OR	95% CI	*p*-Value
High Grade (G3 vs. G1–2)	2.79	(1.42–5.49)	0.003
Deep vs. Superficial	2.14	(0.73–6.25)	0.163
Axial vs. Extremity	2.28	(1.17–4.47)	0.016
R1/R2 vs. R0	0.93	(0.45–1.92)	0.839

Odds ratios (ORs) were derived from a multivariable logistic regression model restricted to patients with local recurrence. The outcome variable was the occurrence of combined local recurrence and metastatic disease versus isolated local recurrence. Covariates included tumor grade, tumor depth, tumor location, and resection margin status. *p*-values are two-sided and considered statistically significant at *p* < 0.05. Abbreviations: OR, odds ratio; CI, confidence interval; G1–G3, tumor grade; R0, microscopically negative margin; R1, microscopically positive margin; R2, macroscopically positive margin.

**Table 3:** Sequence of events in patients with combined local recurrence and metastatic disease.

Sequence of Events	*n* (%)
Metastasis → Local Recurrence	28 (40.0%)
Local Recurrence → Metastasis	24 (34.3%)
Synchronous	18 (25.7%)
**Total**	70 (100%)

The values are presented as the number (percentage) of patients within the combined phenotype (*n* = 70). Event sequences were categorized based on the chronological order of the first documented local recurrence and metastatic disease. “Synchronous” was defined as local recurrence and metastatic disease occurring within 30 days without a clearly distinguishable temporal sequence. Abbreviation: LR, local recurrence.

## Data Availability

The data presented in this study are available on request from the corresponding author.

## Appendix A

The distribution of recurrence phenotypes across major histological subtypes is presented in Table A1. Considerable variation was observed between histological entities. For example, ALT/WDLPS was predominantly associated with the no-event phenotype, whereas leiomyosarcoma more frequently exhibited metastatic disease. However, these findings should be interpreted descriptively, as several subtype-specific groups contained limited numbers of patients and the study was not powered for robust histology-specific analyses.

**Table A1:** Histology-specific distribution of recurrence phenotypes.

Histological Subtype	No. Event	Metastasis Only	LR Only	Both	Total
ALT/WDLPS	85 (79.4%)	2 (1.9%)	18 (16.8%)	2 (1.9%)	107
Undifferentiated/Unclassified Sarcoma	44 (45.4%)	28 (28.9%)	10 (10.3%)	15 (15.5%)	97
Dedifferentiated Liposarcoma	32 (38.1%)	16 (19.0%)	26 (31.0%)	10 (11.9%)	84
Leiomyosarcoma	27 (42.9%)	31 (49.2%)	2 (3.2%)	3 (4.8%)	63
Myxoid Liposarcoma	31 (72.1%)	9 (20.9%)	1 (2.3%)	2 (4.7%)	43
Myxofibrosarcoma	23 (59.0%)	3 (7.7%)	10 (25.6%)	3 (7.7%)	39
Angiosarcoma of Soft Tissue	12 (41.4%)	11 (37.9%)	0 (0.0%)	6 (20.7%)	29
Others	95 (46.1%)	59 (28.6%)	23 (11.2%)	29 (14.1%)	206
**Total**	349	159	90	70	668

The values are presented as *n* (%) within each histological subtype (row percentages). Histological subtypes representing less than 4% of the study cohort were grouped as “Others”. Abbreviations: ALT/WDLPS, atypical lipomatous tumor/well-differentiated liposarcoma; UPS, undifferentiated pleomorphic sarcoma; LR, local recurrence.
